# Location estimation based on feature mode matching with deep network models

**DOI:** 10.3389/fnbot.2023.1181864

**Published:** 2023-06-14

**Authors:** Yu-Ting Bai, Wei Jia, Xue-Bo Jin, Ting-Li Su, Jian-Lei Kong

**Affiliations:** ^1^School of Artificial Intelligence, Beijing Technology and Business University, Beijing, China; ^2^Beijing Laboratory for Intelligent Environmental Protection, Beijing Technology and Business University, Beijing, China

**Keywords:** location estimation, feature extraction, mode classification, deep networks, location system

## Abstract

**Introduction:**

Global navigation satellite system (GNSS) signals can be lost in viaducts, urban canyons, and tunnel environments. It has been a significant challenge to achieve the accurate location of pedestrians during Global Positioning System (GPS) signal outages. This paper proposes a location estimation only with inertial measurements.

**Methods:**

A method is designed based on deep network models with feature mode matching. First, a framework is designed to extract the features of inertial measurements and match them with deep networks. Second, feature extraction and classification methods are investigated to achieve mode partitioning and to lay the foundation for checking different deep networks. Third, typical deep network models are analyzed to match various features. The selected models can be trained for different modes of inertial measurements to obtain localization information. The experiments are performed with the inertial mileage dataset from Oxford University.

**Results and discussion:**

The results demonstrate that the appropriate networks based on different feature modes have more accurate position estimation, which can improve the localization accuracy of pedestrians in GPS signal outages.

## 1. Introduction

In the information age, navigation technology is constantly innovated in national defense and the lives of people and society (Jin et al., 2023). Location estimation and positioning are based on sensors, communication, and electronic control technology to connect resources and information (Dong et al., 2021). Satellite navigation has been the mainstream of location estimation and positioning. However, the navigation signal will be lost due to the special locations, such as viaducts, cities, canyons, and tunnels. Then navigation and positioning cannot be achieved. Other sensors must be used to collect location information, including Wi-Fi, Bluetooth, ultra-wideband, inertial measurement unit (IMU) sensors, etc (Brena et al., 2017). In the assisted positioning systems, the inertial navigation system (INS) has been widely studied and applied due to the signal range, stability, and cost, in which the IMU is the primary sensor (Liu et al., 2021).

The INS simultaneously measures the carrier motion's angular velocity and linear acceleration by gyroscope and accelerometer. Then it solves the real-time navigation information such as 3D attitude, velocity, and carrier position (Poulose and Han, 2019; Chen et al., 2021a). The INS has essential features such as comprehensive information, a fully autonomous mechanism, and easy realization. The INS can work continuously and

stably under various environmental disturbances (Soni and Trapasiya, 2021). The INS first measures the angular velocity information of the carrier by its gyroscope and further calculates the attitude information of the airline. Then the attitude information is used to support the decomposition of the accelerometer measurements. Finally, the detailed navigation information of the carrier is obtained by the transformation of the carrier coordinate system into the navigation coordinate system and then performing the navigation calculation (Cheng et al., 2022). However, the 3D attitude, velocity, and position solved in real-time in INS are achieved by primary and secondary integration of inertial data. The result of such operations will be the measurement error and noise error at the initial operation time, which will be amplified as the operation time increases, eventually leading to an increase in the position error. For this reason, deep learning methods can be considered to avoid generating cumulative errors. Only inertial measurement data and real position information are deemed for end-to-end network training. The accurate estimated position information can be obtained based on the measurement.

With machine learning and deep learning development, various networks are used for navigation and positioning. However, deep networks have different structures and parameters, which treat different data with different effects. Moreover, various situations in life will generate temporal data with different characteristics and differences in the characteristics of different data. Solving the location estimation problem with only one type of deep network is chanllenging. In order to reflect the data characteristics in various modes, the sample entropy is chosen as the metric of the time series complexity. It measures the time series complexity and the probability of generating a new mode when the dimensionality changes. The greater the generating probability, the higher the complexity degree, and the greater the entropy value. The standard deviation and sample entropy of various motion modes are shown in Table 1. It shows that the data presents distinguishing features in different modes. Therefore, this paper focuses on the positioning problem with feature matching and deep learning. We only use the motion data collected by the INS to estimate the position information. Different deep networks are studied and selected according to the data features to avoid the cumulative error problem of traditional inertial navigation. Better positioning accuracy can be achieved in various situations. Firstly, the inertial measurement data are fed into wavelet and one-sided Fourier transform for feature extraction. Secondly, the extracted data are classified by dynamic time regularization and nearest neighbor algorithm. Finally, according to the data class, the data are fed into the matched deep network for position estimation.

**Table 1:** Standard deviation and sample entropy of data in different motion modes.

**Mode**	**Handbag**	**Handheld**	**Pocket**	**Running**	**Slow walking**	**Trolley**
Standard deviation/m	0.9409	1.1345	1.0581	0.9190	1.2245	1.1431
Sample entropy	2.0050	1.9365	2.0394	2.1837	2.1466	2.1048

The rest of this paper is organized as follows. Section 2 describes the existing methods for location estimation. Section 3 describes the proposed location estimation method based on feature mode matching with a deep network model in detail in this paper. Section 4 conducts related experiments on the Oxford inertial mileage dataset and discusses the results. Section 5 concludes the paper and the directions for future research.

## 2. Related works

### 2.1. Traditional navigation and positioning methods

Traditional navigation and positioning techniques are mainly divided into two types: position determination and track projection. Among them, the position determination method relies on the external known position information for positionings, such as satellite navigation, astronomical navigation, and matching navigation. The voyage position projection method is a method to project the following instantaneous position information by measuring the bearing and distance information of the carrier movement under the condition that the initial instaneous position information is known, such as inertial navigation, magnetic compass, and odometry (Duan, 2019). Satellite and inertial navigation are still the most familiar navigation methods to the public at this stage. They are the most widely used, studied, and intensively researched navigation and positioning methods.

IMU sensors used as portable navigation applications in navigation and positioning generally have the characteristics of negligible mass, small size, low cost, and low power consumption (Huang, 2012). However, IMUs have poor performance, and it will be challenging to meet the navigation and positioning needs if they are not limited. The IMU-based positioning technique includes two solutions: the pedestrian dead reckoning (PDR) and the strap-down inertial navigation system (SINS). The PDR is based on step length estimation, which limits the propagation of inertial guidance errors through constrained models such as zero velocity correction. In the literature (Skog et al., 2013), the heading error of the PDR system is effectively eliminated by installing the IMU-based PDR positioning system on both feet. It uses the maximum distance between the two feet to constrain the positioning result of the PDR system. In the literature (Foxlinejicgs, 2005), an indoor pedestrian inertial navigation and positioning system on foot has been proposed. The inertial navigation algorithm divides the pedestrian's gait into zero velocity and motion phases. It reduces the positioning error by estimating and suppressing the inertial sensor error in the zero-velocity interval (Zheng et al., 2016). This algorithm has stability and high accuracy advantages (Zhang et al., 2018). The two solution methods have different principles and advantages.

### 2.2. Positioning methods with IMU

The current navigation method is multi-sensor fusion. A combined GNSS/INS navigation and positioning method is proposed for pedestrian navigation with poor robustness of positioning accuracy and discontinuous position coordinates in indoor and outdoor environments (Wang, 2018; Zhu et al., 2018). In the literature (Liu et al., 2017), Wireless Sensor Networks (WSN) were fused with INS using Kalman to correct the error of firefighters in the forestry field. The advantages of the combined navigation system are reflected in the autonomous inertial navigation when there is no signal from GNSS to ensure the continuity of navigation and the combined navigation when there is a GNSS signal to ensure the navigation accuracy by GNSS constraining the error of INS.

Theoretical studies and experimental validation have been carried out for the filtering methods of GNSS/IMU combined navigation systems. More non-linear filtering algorithms have been proposed successively. The extended Kalman filter algorithm for model error prediction is applied to GNSS/INS combined navigation (Jin et al., 2023). The trace-free Kalman filter algorithm with constrained residuals fuses GPS and PDR positioning information, effectively suppressing the cumulative heading error drift (Niu and Lian, 2017). Particle filtering and robust filtering algorithms can improve the combined navigation filtering algorithm. The information from inertial navigation is fused using particle filtering to improve indoor positioning accuracy (Masiero et al., 2014). A volumetric Kalman filtering algorithm based on gated recurrent unit (GRU) networks has been proposed in the literature (Wang et al., 2022a). The filter innovations, prediction errors, and gains obtained from the filter are used as inputs to the GRU network, and the filter error values are used as outputs to train the network. End-to-end online learning is performed using the designed fully connected network, and the current state of the target is predicted. In Li et al. (2020), a hybrid algorithm based on the GRU and a robust volume Kalman filter is proposed to achieve a combined INS/GPS. It can provide high-accuracy positioning results even when GPS is interrupted. In the literature (Gao et al., 2020), an adaptive Kalman filter navigation algorithm is proposed that adaptively estimates the process noise covariance matrix using reinforcement learning methods. A sideslip angle estimation method combining a deep neural network and a non-linear Kalman filter has been proposed in the literature (Kim et al., 2020). The estimation of the deep neural network is used as a new measure of the non-linear Kalman filter, and its uncertainty is used to construct the adaptive measurement covariance matrix. The effectiveness of the algorithm is verified by simulation and experiment. According to the actual engineering requirements, when one of the system's subsystems does not work, this subsystem is removed in the fusion process, which improves the system's stability and is fully applied in various practical projects. The combined navigation technology mainly uses the positioning characteristics of INS and GNSS to combine them effectively and take advantage of their respective advantages to accomplish navigation tasks (Wu et al., 2020).

However, when GNSS is affected by the external environment, its poor anti-jamming capability makes it impossible to properly combine GNSS and INS technologies for navigation, and only INS technologies combined with depth networks can be relied on for navigation and positioning to compensate for the lack of GNSS. The literature (Yang, 2019) divides the positioning process into offline and online. In the offline process, the DNN model is trained using the signals from the signal towers, while in the online phase, the positioning process is implemented using the existing model. The literature (Wang, 2019) converts the visual information into one-dimensional landmark features using a convolutional neural network (CNN) based landmark detection model. In contrast, the wireless signal features are extracted using a weighted extraction model, and finally, the position coordinates are estimated using a regression method. The literature (Cheng et al., 2021) considers the continuity of wireless signals in the time domain during localization. It uses long short-term memory (LSTM) and temporal convolutional network (TCN) to extract features from signal sequences and calculate the localized object's position. A new AI-assisted approach for integrating high-precision INS/GNSS navigation systems is proposed in the literature (Zhao et al., 2022). Position increments during GPS interruptions are predicted by CNN-GRU, where CNN extracts multidimensional sequence features rapidly, and GRU models the time series for accurate positioning. In the literature (Liu et al., 2022), a GPS/INS neural network (GI-NN) is proposed to assist INS. The GI-NN combines CNN and GRU to extract spatial features from IMU signals and track their temporal features to build a relational model and perform a dynamic estimation of the vehicle using current and past IMU data. This paper will focus on the different effects of different networks when dealing with different data sets, and the adapted networks can improve the localization accuracy of the corresponding data.

Accurate positioning is difficult to achieve in complex environments, and the fusion of multiple technologies will solve this challenge. Neural networks are begin to significantly impact inertial navigation, where data feature analysis has been a vital issue.

## 3. Location estimation based on feature mode matching with deep network models

### 3.1. Estimation framework of feature extraction and deep networks

**Figure 1:**
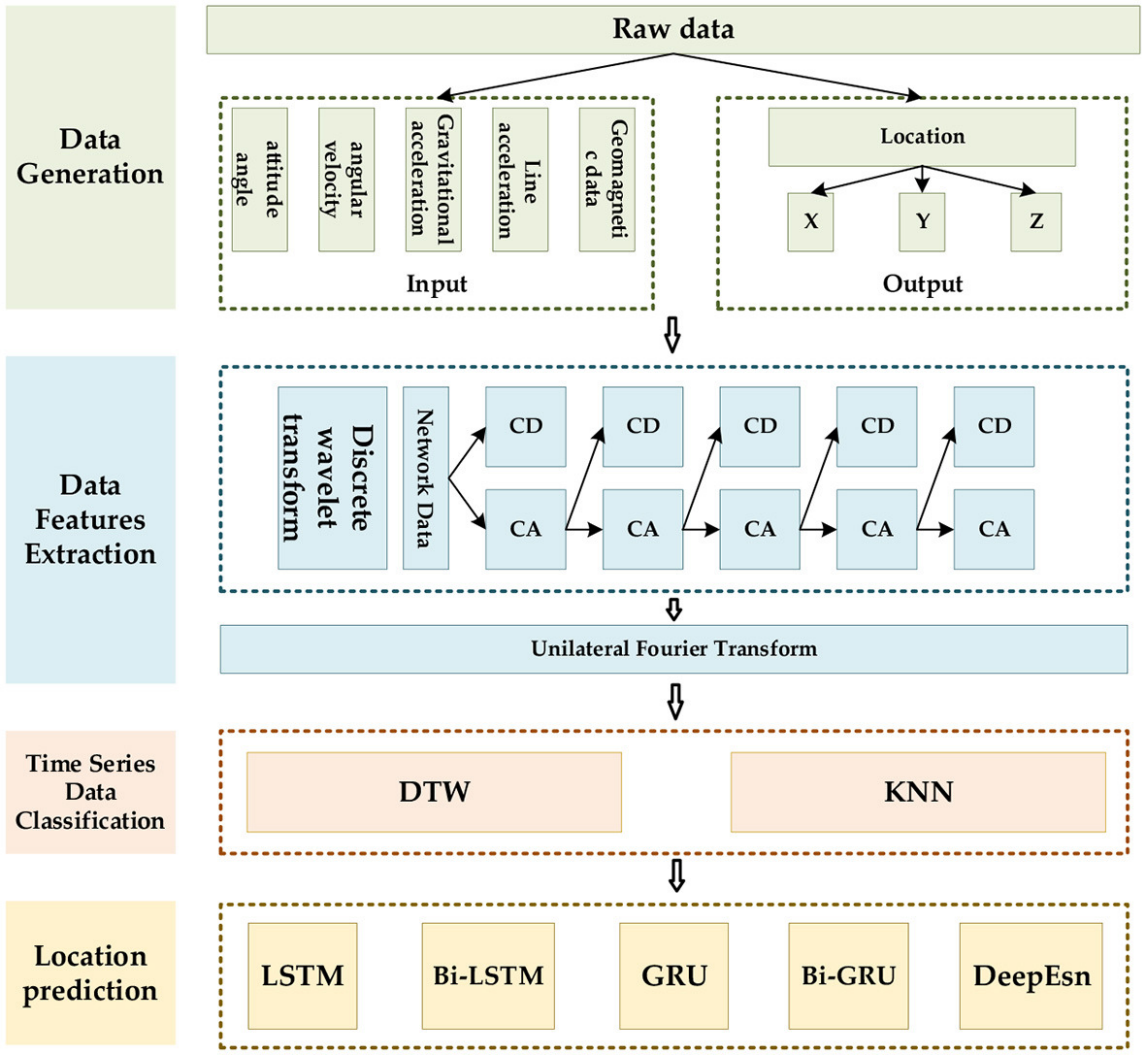
The framework of the location estimation is based on deep network matching mode features.

The data feature should be extracted first for accurate location prediction for various motion modes. We use the discrete wavelet, and Fourier transforms for different data, then classify and identify the extracted features, and finally select the deep network models that are compatible with the features. The networks are selected from the typical LSTM, bi-directional long short-term memory (Bi-LSTM), GRU, bi-directional gated recurrent unit (Bi-GRU), and deep echo state network (DeepEsn) networks. The structure of the location adaptive estimation method for the automatic matching of deep networks is shown in Figure 1.

### 3.2. Feature extraction and classification

Time-series data are recorded in chronological order over a specified period. All data in the same data column are of the same caliber and are comparable (Wang et al., 2021). Since time-series data are usually accurate records of system information, they reflect the trend of system changes over time by describing the state of things or phenomena, which often implies the potential laws and characteristics of the system (Kong, J. et al., 2023). Therefore, uncovering and exploiting these laws and characteristics through studying time series data is an effective means of bringing the value of time series data into play (Kong, J.-L. et al., 2023). It is also possible to classify the time series data by comparing the laws and characteristics in the time series data. Different categories of time series data will correspond to different data processing methods so that the characteristics of the data can be used more effectively.

**Figure 2:**
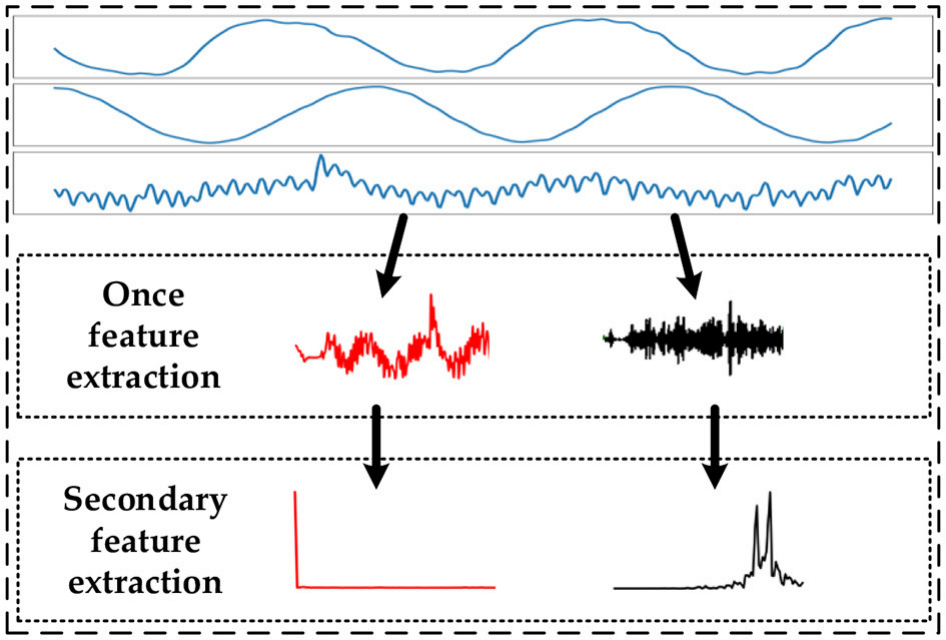
Schematic diagram of data feature extraction.

The data features are extracted by performing two sequential processes on the data using the discrete wavelet transform and the Fourier transform. The first feature extraction is a discrete wavelet transform of the time-series data, and the second feature extraction is a Fourier transform of the first extracted feature sequence. The feature extraction process is shown in Figure 2.

The wavelet transform has the properties of local variation, multiresolution, and decorrelation (Vidakovic and Lozoya, 1998). It translates the data at different scales to obtain wavelet coefficients. The discrete wavelet transform (DWT) in wavelet transform decomposes the data by high-pass and low-pass filters to produce an approximate component of approximate (CA) and component of detail (CD), respectively (Deng, 2021; Wang et al., 2022b). When performing a multi-order DWT, the CD is processed using a high-pass filter, while CA will continue to be decomposed. The multi-order decomposition is used to correct the high-frequency information in the data and effectively extract the data features. The discrete wavelet transforms equation and the DWT algorithm expressions are shown in equations (1) and (2), respectively.


(1) $$\begin{array}{l} \text{f}(\text{t})\text{=}\sum_{\text{j=-}\infty }^{\text{j}}\left[\sum_{\text{k=-}\infty }^{\infty }{\text{d}}_{\text{j},\text{k}}\;{\text{ϕ}}_{\text{j},\text{k}}(\text{t})\text{+}\sum_{\text{k=-}\infty }^{\infty }{\text{c}}_{\text{j},\text{k}}\text{ ϕ}(\text{t})\right] \end{array}$$

where $\sum_{\text{k=-}\infty }^{\infty }{\text{c}}_{\text{j},\text{k}}\text{ϕ}\left(\text{t}\right)$ is approximate data (low-frequency). $\sum_{\text{k=-}\infty }^{\infty }{\text{d}}_{\text{j},\text{k}}{\text{ϕ}}_{\text{j},\text{k}}\left(\text{t}\right)$ is the detail data(high-frequency). ϕ_*j,k*_(*t*) is the basic wavelet function. ϕ(t) is the scale function.


(2) $$\begin{array}{l} {\text{A}}_{\text{i}}\text{f}\left(\text{t}\right)={\text{A}}_{\text{i}+1}\text{f}\left(\text{t}\right)+{\text{D}}_{\text{i}+1}\text{f}\left(\text{t}\right) \end{array}$$

where A_i_*f*(*t*) is the low-frequency part of the wavelet decomposition of the first layer. A_i+1_*f*(*t*) and D_i+1_*f*(*t*) are the low frequency part and high frequency part of the next layer of decomposition, respectively.

The Fourier transform is a standard method for analyzing signals. The process converts a continuous signal that is non-periodic in the time domain into a continuous signal that is non-periodic in the frequency domain. The same principle can be used to analyze and process time-series data, and the Fourier transform is shown in equation (3).


(3) $$\begin{array}{l} \text{F}\left(\omega \right)\text{=F}\left(\text{f}\left(\text{t}\right)\right)\text{=}\int_{\text{-}\infty }^{\text{+}\infty }\text{f}\left(\text{t}\right){\text{e}}^{\text{-i}\omega \text{t}}\text{dt} \end{array}$$

where F(ω) is the image function of f(t). f(t) is the original image function of F(ω).

Classification of temporal data is mainly divided into benchmark methods, which use feature similarity as a determination. Traditional methods classify data by underlying modes and features, and deep learning classification methods. Classification by deep learning methods performs very well on image, audio, and text data and can quickly update data using batch propagation (Jonathan et al., 2020). However, they are unsuitable as general-purpose algorithms because they require large amounts of data. Classical machine-learning problems are usually better than tree collections. Moreover, they are computationally intensive during training and require more expertise to tune the parameters. The Oxford Inertial Mileage dataset is characterized by various types of time-series data and a small number of data sequences. Compared to deep learning methods that require architecture and hyperparameter tuning, traditional methods that determine the similarity of classification features are more straightforward and faster and can achieve good classification results.

**Figure 3:**
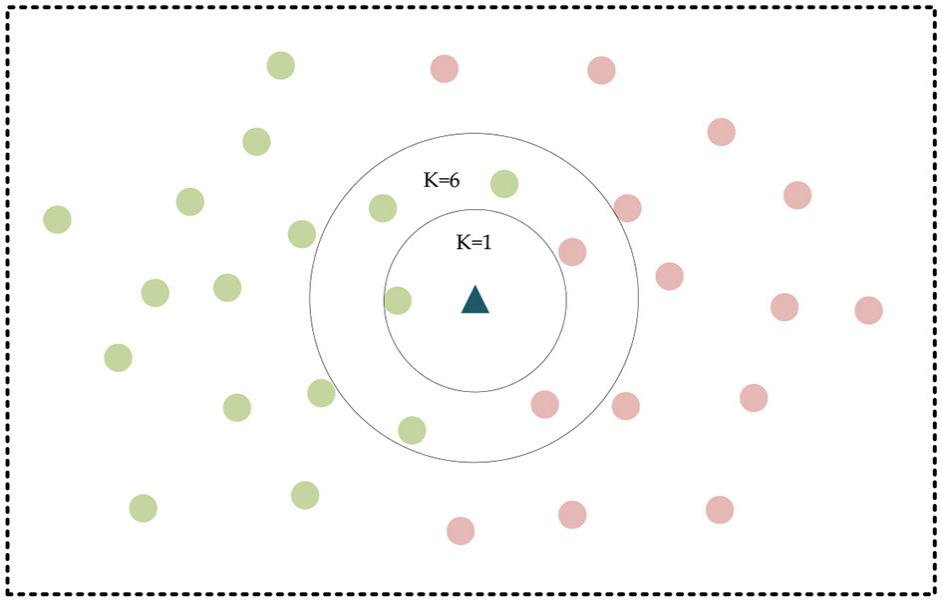
KNN schematic diagram.

It usually classifies features using Euclidean distance and dynamic time warping (DTW). They are set as the similarity measure by calculating the distance between the original or temporal data after feature representation (Pimpalkhute et al., 2021). Then it uses the nearest neighbor classifier for classification. This similarity metric-based method for classifying temporal data is simple in principle and structure, easy to implement, and is considered the benchmark method for classifying temporal data. The K-Nearest Neighbors (KNN) algorithm has been the simple and typical classification algorithm. In KNN, when a new value *x* is predicted, the class to which *x* belongs is determined based on its class from the nearest K points (Zhang, 2021). The KNN schematic is shown in Figure 3, in which the green and red dots represent the two categories, and the triangular points are the points to be classified.

The distance calculation is usually chosen as the Euclidean distance with the equation.


(4) $$\begin{array}{l} \text{d = }\sqrt{{\left({\text{x}}_{\text{1}}{\text{− y}}_{\text{1}}\right)}^{\text{2}}\text{+}{\left({\text{x}}_{\text{2}}{\text{− y}}_{\text{2}}\right)}^{\text{2}}\text{+}\cdots \text{+}{\left({\text{x}}_{\text{n}}{\text{− y}}_{\text{n}}\right)}^{\text{2}}}\\ \text{ =}\sqrt{\sum_{\text{i}}^{\text{n}}{\left({\text{x}}_{\text{i}}{\text{− y}}_{\text{i}}\right)}^{\text{2}}} \end{array}$$

where *x* and *y* are coordinates in the two-dimensional plane, and the subscripts are the ordinal numbers of the data points.

The dynamic time regularization algorithm is a proposed metric between sequences for time-series data. The DTW algorithm finds the best correspondence between two observed sequences by regularizing the time dimension with certain constraints. Therefore, DTW is suitable for classifying sequences with different frequencies or phases. DTW uses the idea of dynamic programming to calculate the optimal path between two sequences, where the dynamic transfer equation is as follows.


(5) $$\begin{array}{l} \text{D}\left(\text{i},\text{j}\right)=\text{Dist}\left(\text{i},\text{j}\right)+\text{min}\left[\text{D}\left(\text{i}-1,\text{j}\right),\text{D}\left(\text{i}-1,\text{j}\right),\text{D}\left(\text{i}-1,\text{j}\right)\right] \end{array}$$

where D(i, j) is the coordinate of the distance matrix, Dist(i, j) is the calculated Euclidean distance, and D(i − 1, j), D(i, j − 1), and D(i − 1, j − 1) are the lower left 3 elements of D(i, j), respectively.

### 3.3. Feature model matching with deep networks

The data in different modes can be selected to fit the depth network according to their different feature information. From parts 3.1 and 3.2 of this paper, different mode features can be extracted and distinguished effectively, then according to the different mode features to match the depth network that fits with the features, which can better perform the performance of network estimation. By mathematically analyzing the time-series data features, the features can be characterized by the mean, variance, skewness, and kurtosis of the data features. The mean and variance can reflect the overall trend in the data set, and the skewness and kurtosis can reflect the local details in the data set. Equation (6) shows the overall form of the evaluation data features, f_a_ is the evaluation in a mode which contains p_mean_, p_variance_, p_peakedness_ and p_skewness_, representing the mean, variance, kurtosis, and skewness of the feature data, respectively.


(6) $$\begin{array}{l} {\text{f}}_{\text{a}}=\text{f}\left({\text{p}}_{\text{mean}},{\text{p}}_{\text{variance}},{\text{p}}_{\text{peakedness}},{\text{p}}_{\text{skewness}}\right) \end{array}$$

The networks selected in this paper include LSTM, Bi-LSTM, GRU, Bi-GRU, and DeepEsn. LSTM networks consist of forgetting, input, and output gates, which can handle longer data sequences and solve the problems of gradient disappearance and gradient explosion problems. The GRU network is a suitable variant of the LSTM network, and its structure is more concise than the LSTM. The two-way network can correlate the next-moment state information and the previous-moment state information to estimate the output with the previous and future states. Finally, as an improved ESN network, the DeepEsn develops from a single reserve pool to a deep learning network consisting of a multi-layer reserve pool structure in series. The characteristics of leaky integral-type neurons in each reserve pool will effectively improve the memory of network history information. The networks are selected according to the trend information and detailed information of the data features according to the characteristics of the five deep network models selected in this paper. The network selection of data modes is shown in Table 2.

**Table 2:** Network selection regarding the feature mode matching.

**Mode category**	**Sample entropy**	**Decomposition results graph**	**Deep network to be selected**	**Remarks**
Handbag	2.005	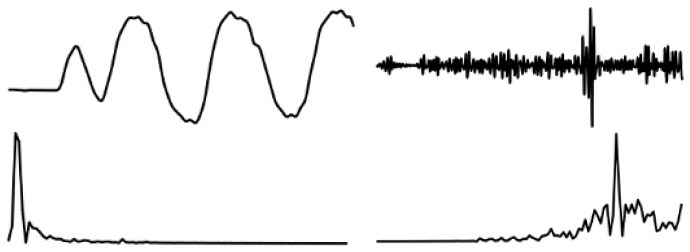	Bi-LSTM GRU	The data has a clear cyclical trend, with a dense and small magnitude of detailed trends.
Handheld	1.936	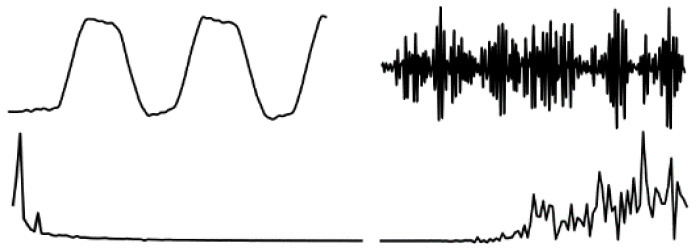	DeepEsn LSTM	The data has a clear cyclical trend, and the detailed features are intensive and cyclical.
Pocket	2.309	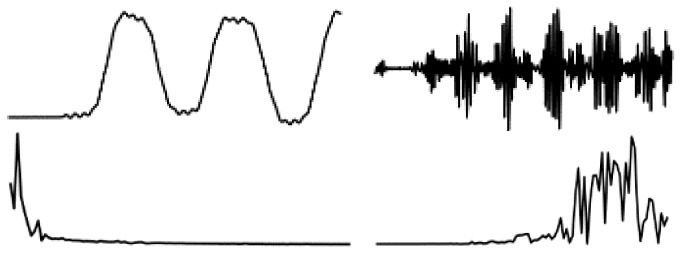	DeepEsn	The data has a clear cyclical trend, and the detailed features are intensive and cyclical.
Running	2.183	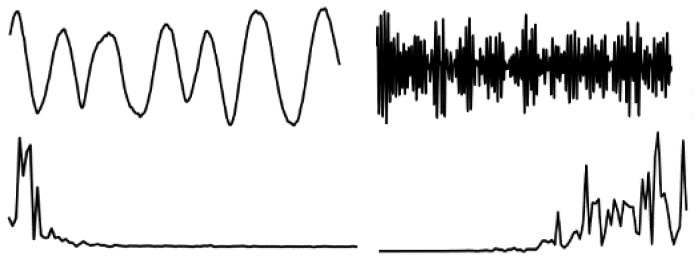	LSTM GRU	The data has a clear cyclical trend and segmented period, with intensive and detailed features.
Slow walking	2.146	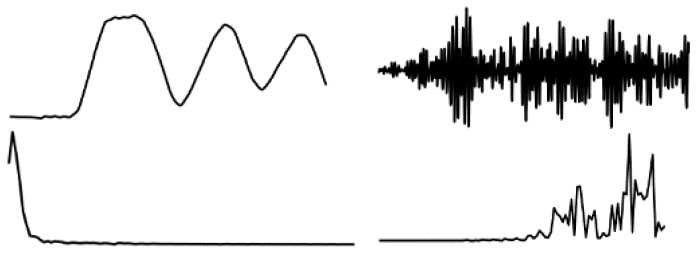	DeepEsn Bi-GRU	The data has a clear cyclical trend, and the detailed features are intensive and cyclical.
Trolley	2.104	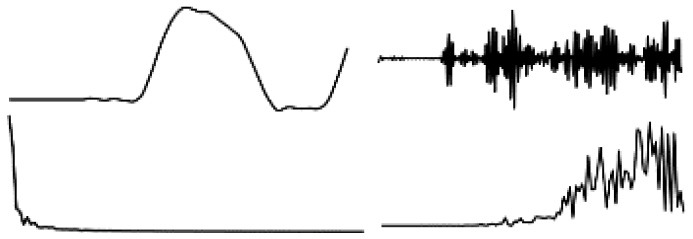	Bi-GRU	The data has a clear and long cyclical trend; sudden changes dominate the detailed features.

**Figure 4:**
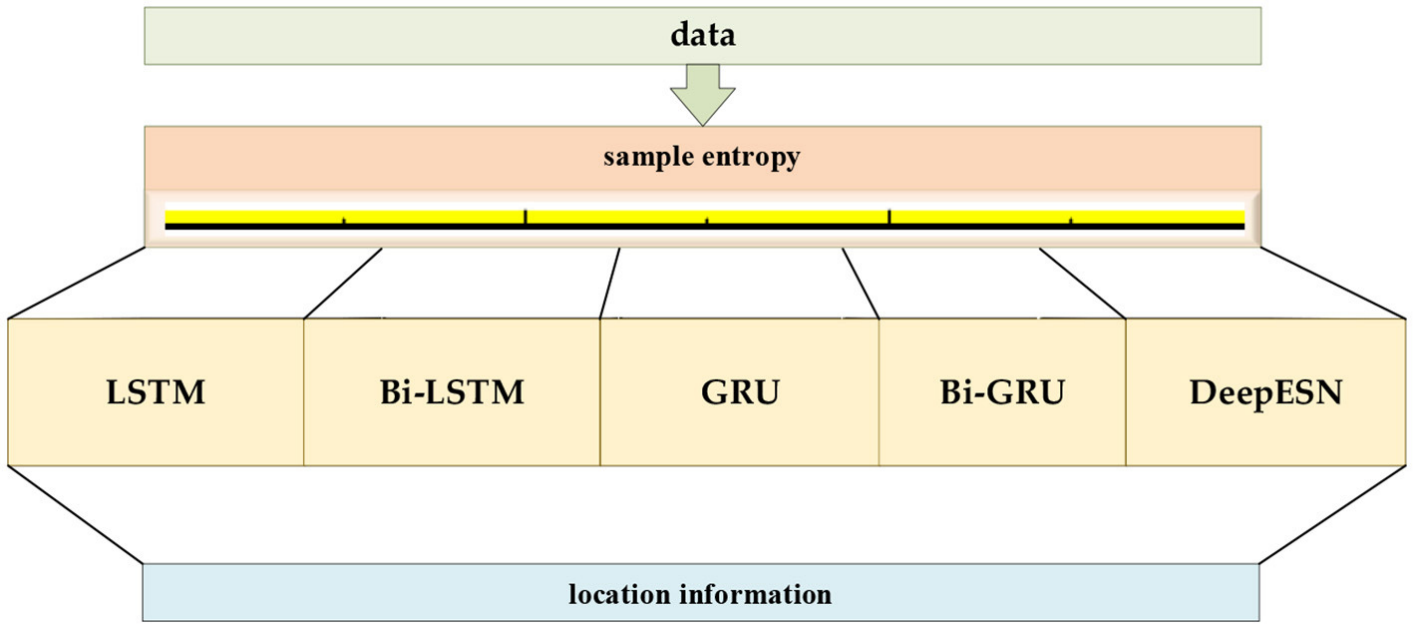
Algorithm flow of selecting networks for different mode data.

The basis for selecting networks for different modes is based on different temporal complexity between data to reflect the data characteristics of different modes to determine which network should be selected. The temporal complexity can be judged based on the size of the sample entropy to determine the network for location estimation and achieve location estimation. The sample entropy has a direct relationship with the complexity of the data. The higher the sample entropy, the higher the complexity of the data, and the more the deep network with higher processing data is needed to process to achieve better results. The deep networks selected in this paper contain LSTM, GRU, Bi-LSTM, Bi-GRU, DeepESN networks. GRU is an improved network of LSTM network, but the prediction ability of the network is similar, only in the training speed of the model is improved; Bi-LSTM and Bi-GRU networks as improved networks of LSTM, GRU, the network model from only Bi-LSTM and Bi-GRU networks are improved networks of LSTM and GRU. The network models use information from the forward direction to the forward and backward movement, which makes the network models process more complex data and improve prediction accuracy; DeepESN networks have more complex layers than other network models and can handle more complex data. Therefore, the selection of the deep network is directly related to the complexity of the data. The higher the complexity of the data, the higher the sample entropy, and the deep network should be more complex. The flow chart of network selection for different modes is shown in Figure 4.

Figure 4 shows the algorithm flow of selecting networks for different data modes. The original data are first subjected to the calculation of sample entropy. The corresponding network is chosen according to the magnitude of the sample entropy, and finally, the selected position estimation is achieved using the selected depth network.

## 4. Experiment and result

In this section, we use the Oxford Inertial Ranging Dataset (OxIOD), classify the data based on its features, and select compatible networks from LSTM, Bi-LSTM, GRU, Bi-GRU, and DeepEsn network models based on various types of features. The PDR is also set as the baseline model, which uses the movement speed and forward direction to infer the positioning process. Finally, extensive experiments are conducted to verify the appropriateness of the network selection from the estimated network results.

### 4.1. Data sets and experiment setting

In this paper, we use the OxIOD dataset, in which ground truth data for indoor walking is collected using the Vicon optical motion capture system, which is known for its high accuracy (0.01 m in position and 0.1 degrees in direction) in target localization and tracking (Chen et al., 2020; Kim et al., 2021). The IMU sensors on smartphones. It includes data from four off-the-shelf consumer phones and five different users and data from different locations and motion states of the same pedestrian, including handheld, pocket, handbag, and stroller data in a normal walking motion, slow walking, and running (Markus et al., 2008; Chen et al., 2021b). The raw inertial measurements were segmented into sequences with a window size of 200 frames (2 s) and a step length of 10 frames. OxIOD's data is extensive and has highly accurate actual values, making it suitable for deep learning methods. At the same time, the dataset contains a wide range of human movements that can represent everyday conditions, providing greater diversity.

For each type of data, different divisions were performed. The training set is 7 sequences for handbag data, 10 sequences for pocket data, 20 sequences for handheld data, 6 sequences for running data, 7 sequences for slow walking data, and 12 sequences for cart data. The test set is the rest of the sequences. The input of each experiment below is 15 data items of sensors in the dataset, and the output is 13 data items, namely, changing displacement, heading angle, changing heading angle, average speed, speed of heading angle, changing the speed of heading angle, translation.x, translation.y, translation.z, rotation.x, rotation.y, rotation.z, and rotation.w. This paper will focus on the output position information translation.x and translation.y for experimental study.

### 4.2. Feature extraction and classification

**Figure 5:**
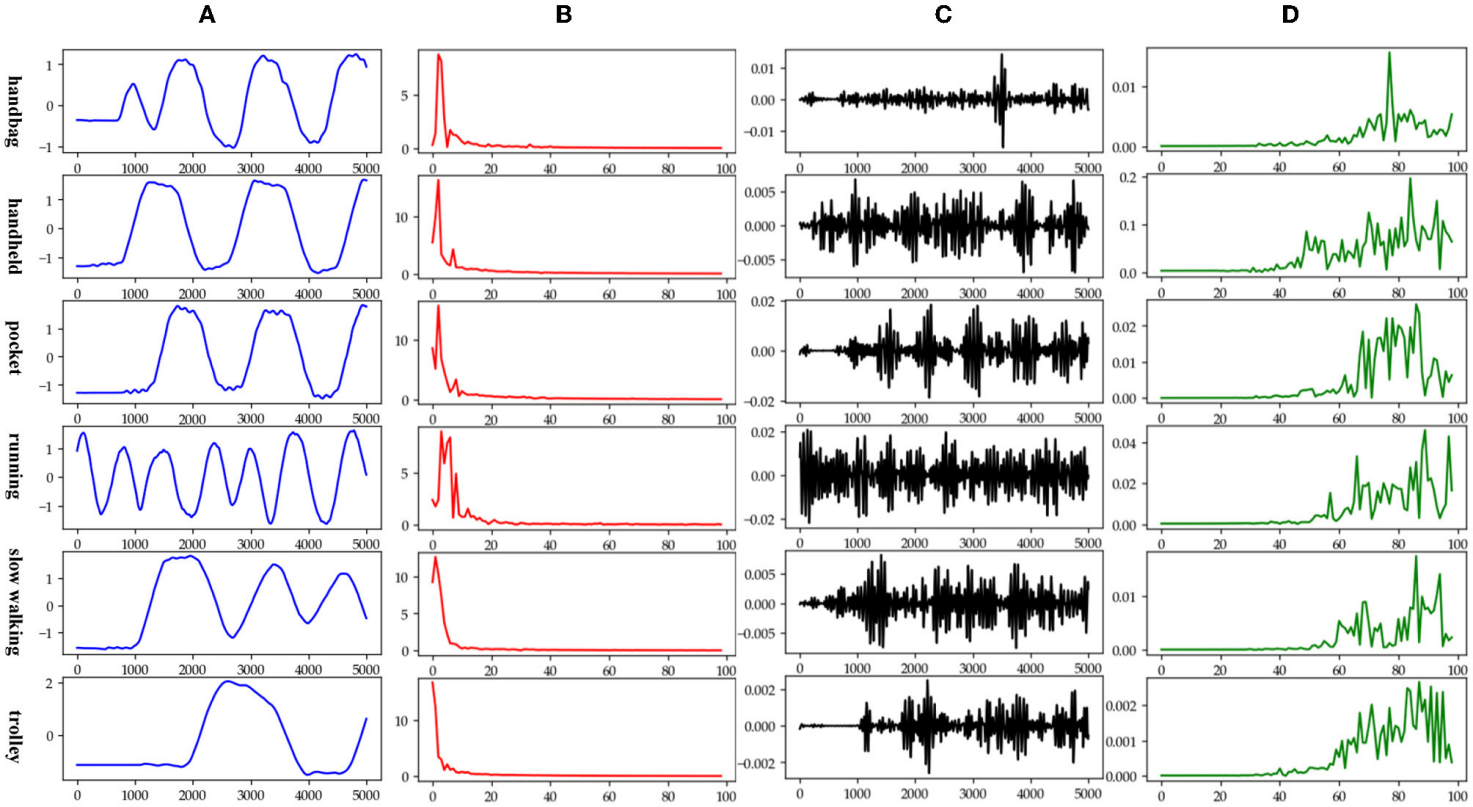
Data decomposition results for each motion mode: **(A–D)** are the unilateral Fourier variation of CA and CA after decomposition of the fifth-order discrete wavelet transform, and the unilateral Fourier variation of CD and CD after decomposition of the fifth-order discrete wavelet transform, respectively.

The data in various modes have different data characteristics. We can qualitatively find the individual characteristics and assign the appropriate deep network model through data decomposition and feature extraction. Wavelet decomposition can decompose signals at different scales, and the choice of different scales can be determined according to different objectives. Wavelet decomposition achieves feature extraction by decomposing the low-frequency and high-frequency features of the data. In this paper, the db5 wavelet, which is widely used and has a better processing effect, is chosen as the wavelet base, and the number of decomposition layers is chosen as 5. The decomposition results of the data of various modes are shown in Figure 5.

The subplot in Figure 5 represents the results of the decomposition of handbag, handheld, pocket, running, slow walking, and trolley mode data in each of the 6 rows of subplots from top to bottom. The figure's four subplots from left to right are columns A, B, C, and D. The blue and black line subplots in columns A and C of the figure show the CA and CD after decomposition of the fifth-order discrete wavelet transform, which has a data volume of 5,000, allowing differences in frequency, amplitude, and other relevant information to be observed. However, the results cannot be directly observed quantitatively. The figure's red and green line plots in columns B and D are the spectrum plots of CA and CD with unilateral Fourier variation, respectively. Based on the spectrum plots of CA and CD, various modes can be effectively distinguished, and the corresponding deep network model. The Bi-LSTM network is selected for the data in the handbag mode, while the Bi-LSTM network is selected for the data in the handheld, pocket, running, or slow walking modes. The DeepEsn, LSTM, and Bi-GRU networks are selected for position estimation for the trolley mode. The number of reserve layer layers in the DeepEsn network and the number of neurons in the reserve layers in the DeepEsn network are chosen separately according to the situation. The results of the data feature representation of each mode are shown in Table 3.

**Table 3:** Data characteristics of each motion mode.

**Mode**	***p*_*mean*_**	***p*_*variance*_**	***p*_*peakedness*_**	***p*_*skewness*_**
Handbag	0.4302	1.59	33.633	5.619
Handheld	0.7691	4.04	40.954	6.008
Pocket	0.8235	3.98	33.098	5.292
Running	0.5978	2.66	16.142	4.015
Slow walking	0.6053	3.99	22.027	4.672
Trolley	0.6772	4.99	34.816	5.727

**Figure 6:**
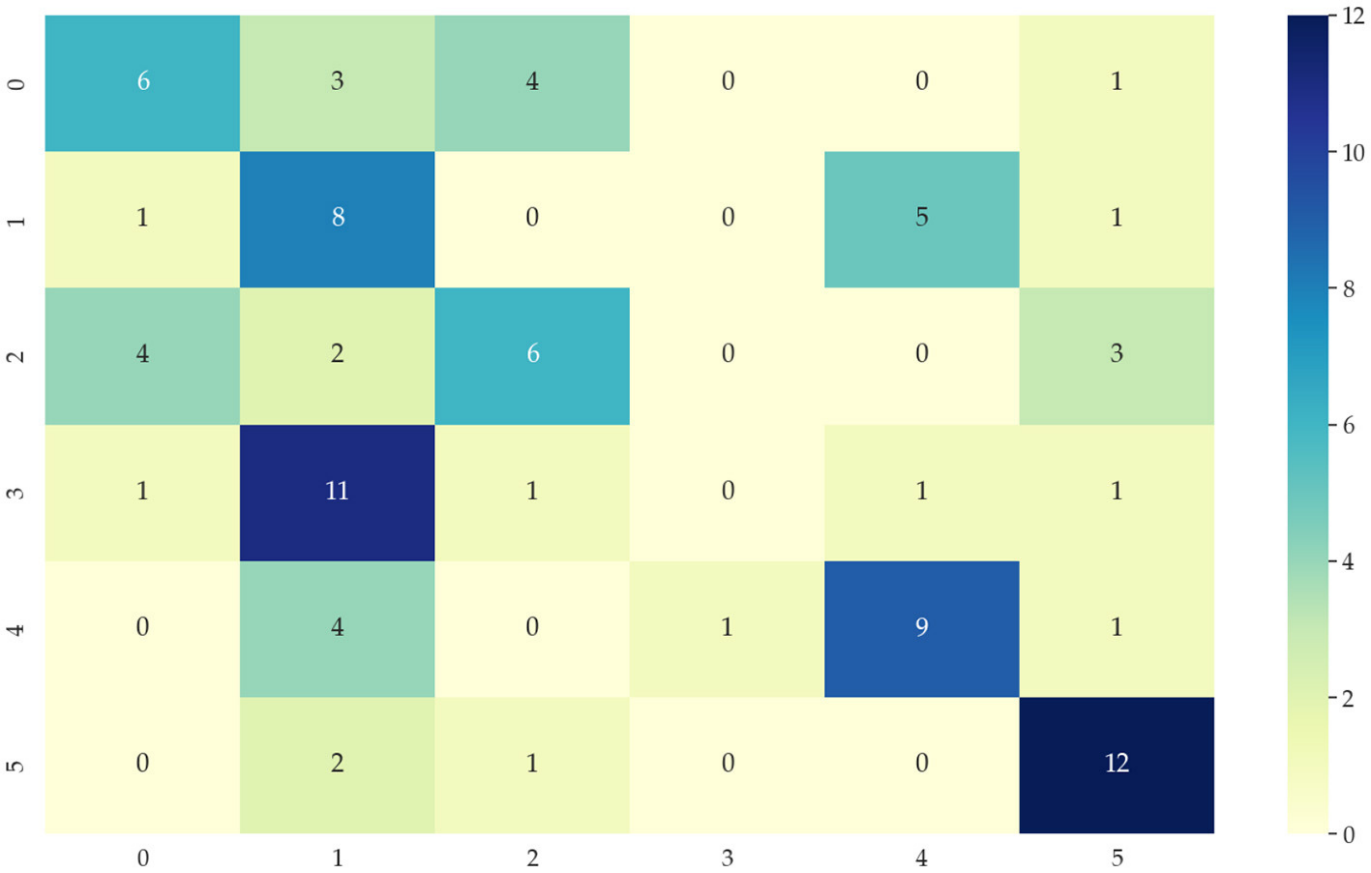
Classification confusion matrix from DTW + KNN algorithm of original data.

**Figure 7:**
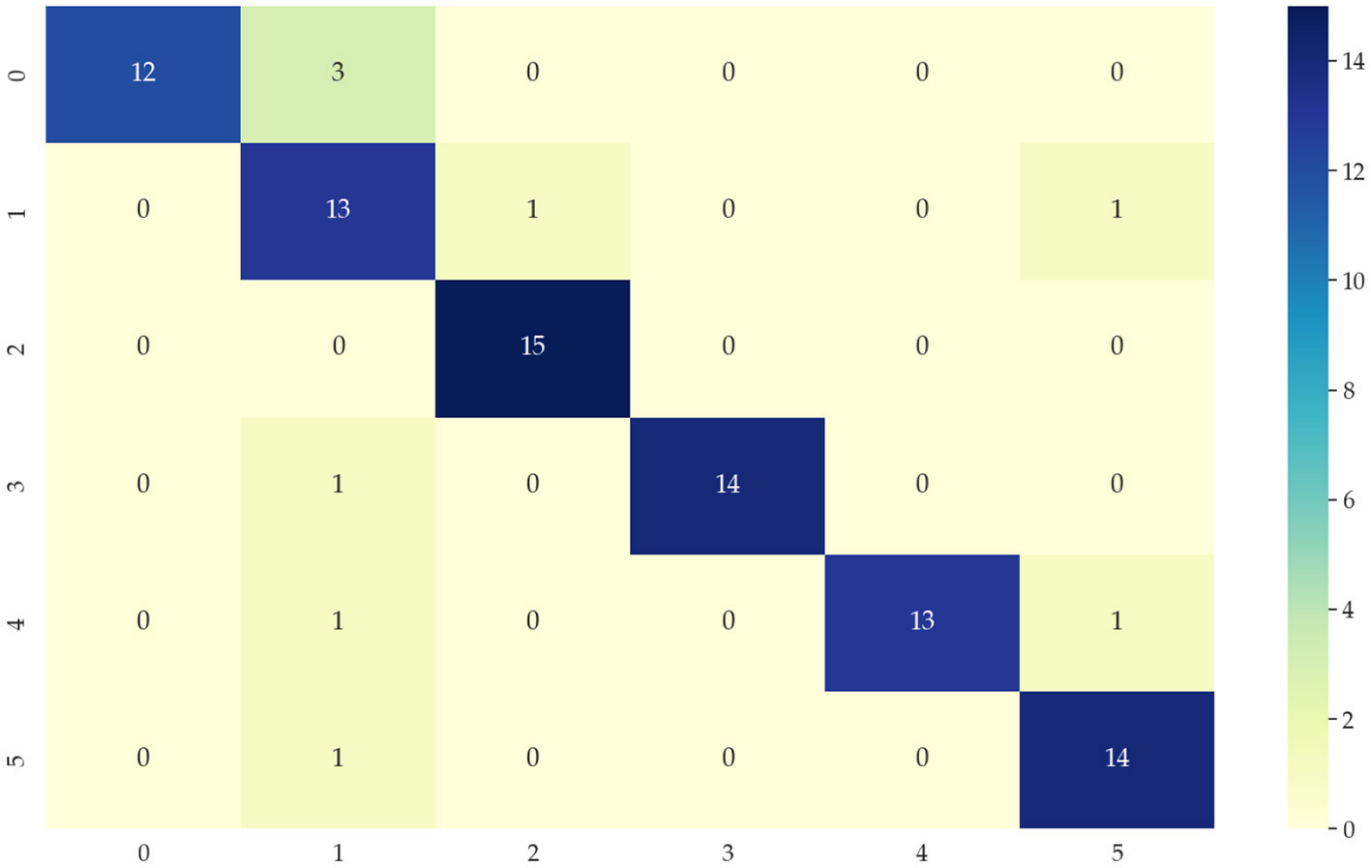
Classification confusion matrix after data feature extraction.

Identifying data types for classification means that the input temporal data is used to correctly distinguish which category of the six modes mentioned in 4.1 is identified to correctly select the appropriate network model. This paper chooses the KNN algorithm of the dynamic time regularization (DTW) algorithm for classification. However, the direct recognition and classification of the original data do not extract the hidden features in the data well, and its classification results are poor, as shown in Figure 6. Therefore, we need to identify and classify the sequences after extracting the features to improve the accuracy. After decomposing the features extracted by the 4.2 part of the data, the accuracy of the classification results can reach 90%, and the classification results are shown in Figure 7. 0, 1, 2, 3, 4, and 5 in the horizontal and vertical coordinates correspond to 6 types of mode data.

### 4.3. Location estimation in different modes

**Figure 8:**
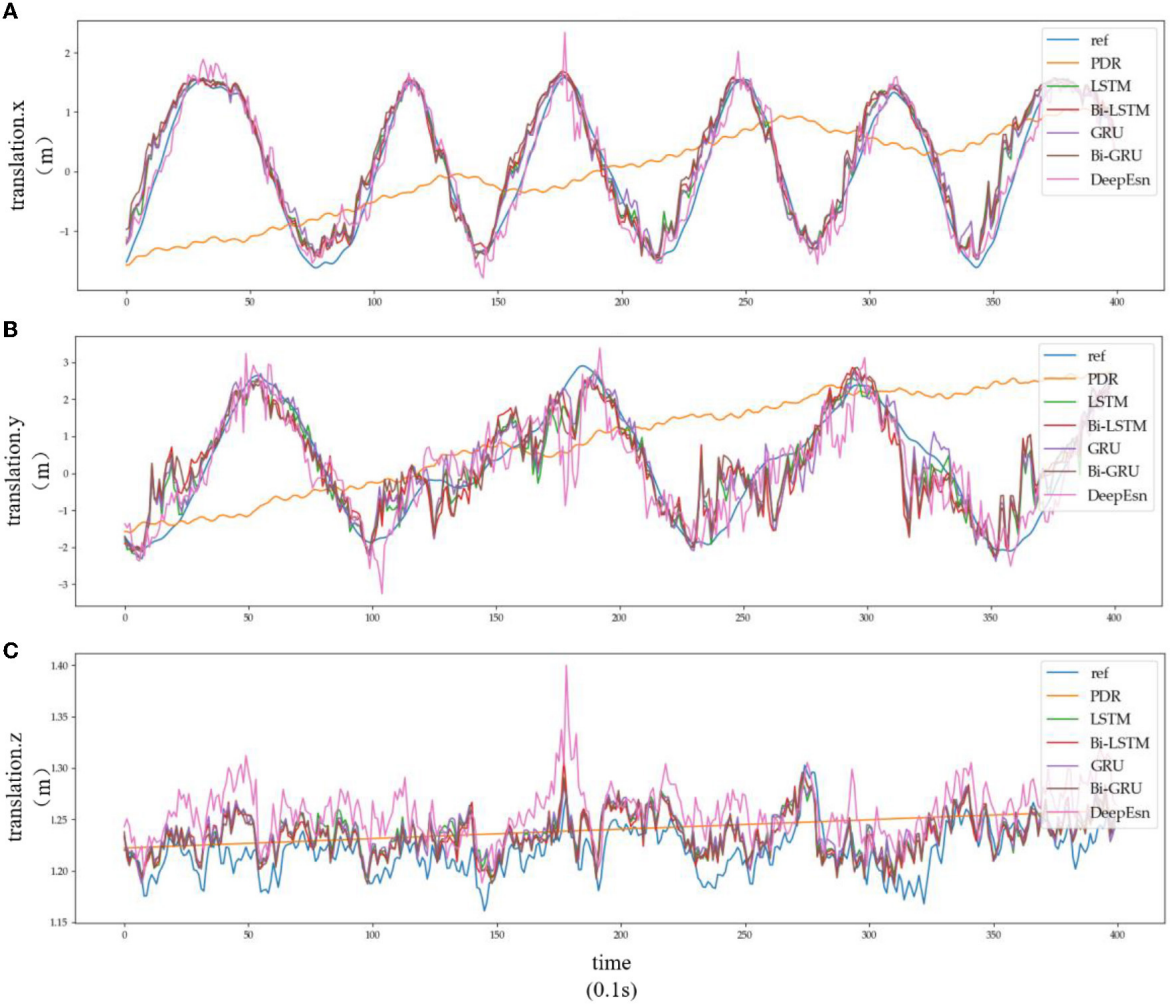
Location estimation results of different deep networks in running mode: **(A–C)** is the Location estimation results of translation.x, translation.y, and translation.z in running mode, respectively.

**Figure 9:**
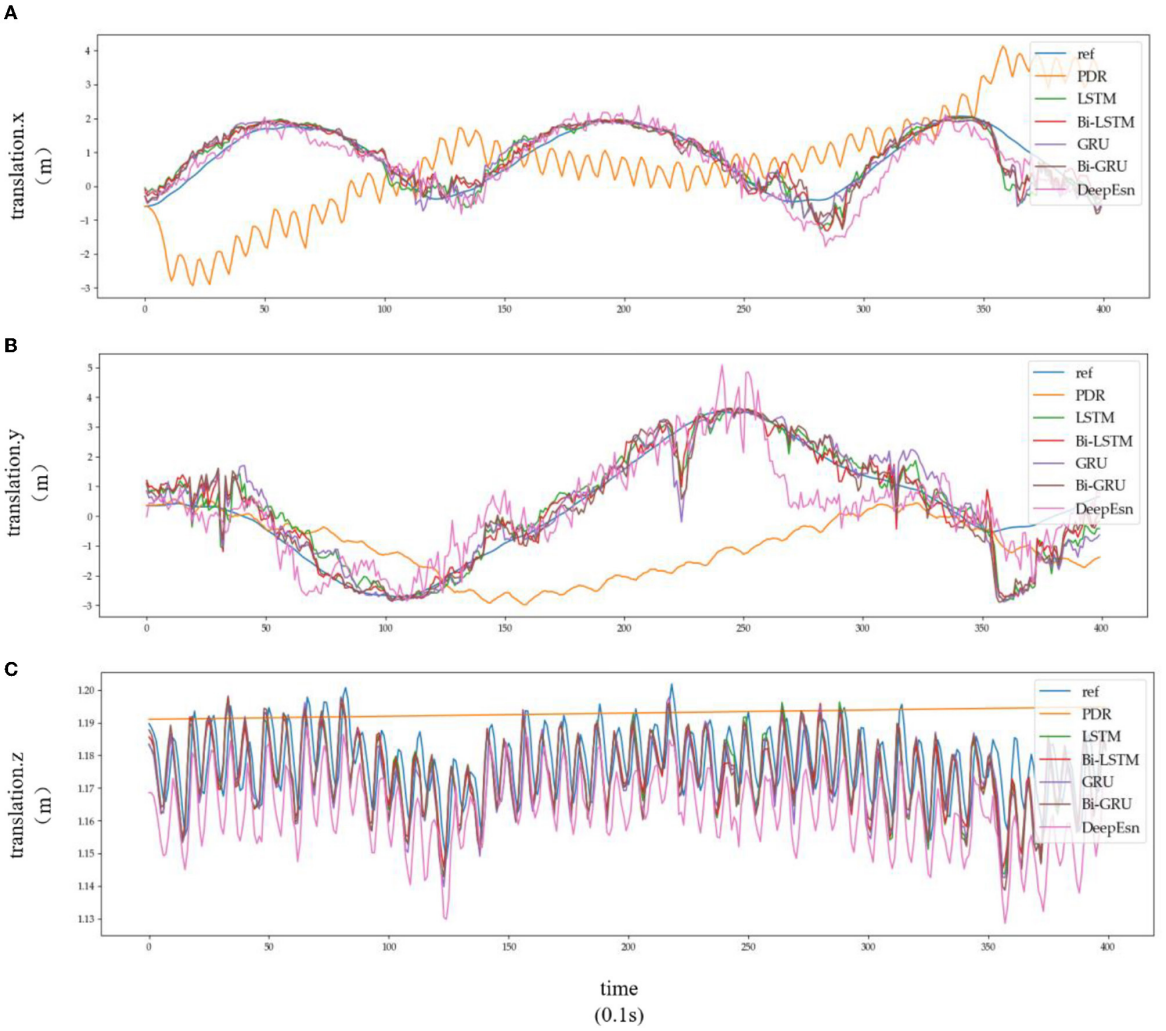
Location estimation results of different deep networks in slow walking mode: **(A–C)** is the Location estimation results of translation.x, translation.y, and translation.z in slow walking mode, respectively.

This paper uses the OxIOD dataset and attempts to better solve the pedestrian inertial navigation problem using different deep neural networks depending on the model. This paper uses LSTM, Bi-LSTM, GRU, and Bi-GRU network models with two-layer networks with 128 and 64-dimensional hidden states, respectively. In contrast, DeepEsn networks use the best network structure with the number of reserve layers ranging from 1 to 7 and the number of neurons in the reserved layer ranging from 500 to 750. The models were trained using detailed split training sets for the four attachment categories mentioned above, namely, handheld (20 sequences), pocket (10 sequences), handbag (7 sequences), cart (12 sequences), running (6 sequences), and slow walking (7 sequences). The different split types of datasets were put into the neural network for training, and the input data remained IMU sensor data. The output results were selected with the location based translation.x, translation. y and translation.z data for viewing and comparison with the baseline model; the results are shown in Figures 8, 9, and Table 4. The detailed RMSE, MSE, R, and R2 evaluation metrics are shown in Table 5. Figures 8, 9 show the estimation results of the three-way location coordinates over time for the predictions of different networks on running and slow walking data, respectively, in terms of position information. The LSTM, Bi-LSTM, GRU, and Bi-GRU networks in the article experiments are all 2-layer structures, and the number of neurons per layer is 32. For the DeepESN network, the number of reserve pool neurons ranges from 400 to 600, and the number of layers ranges from 1 to 7, and the optimal result is chosen as the final structure of DeepESN.

**Table 4:** Evaluation metric of EvaMe in the running and slow walking mode data.

**Mode**	**PDR**	**LSTM**	**Bi-LSTM**	**GRU**	**Bi-GRU**	**DeepEsn**
Running	0.89214	**0.25351**	0.28212	0.27012	0.30051	0.36852
Slow walking	2.12199	0.30596	0.30891	0.3048	**0.29656**	0.446

The bold values indicate the best evaluation metrics for the various methods in running and slow walking mode, respectively.

**Table 5:** Evaluation metrics for each network in the running and slow walking modes.

**Mode**			**PDR**	**LSTM**	**Bi-LSTM**	**GRU**	**Bi-GRU**	**DeepEsn**
Running	translation.x	RMSE	1.227133	0.365245	0.375894	0.369127	0.423125	0.333949
		MSE	1.505855	0.133404	0.141296	0.136255	0.179035	0.111522
		R	0.077798	0.955243	0.949109	0.950859	0.941615	0.941075
		R2	−1.836009	0.857052	0.848594	0.853997	0.808156	0.880499
	translation.y	RMSE	2.003978	0.735532	0.822200	0.790001	0.849290	0.813939
		MSE	4.015928	0.541007	0.676013	0.624102	0.721294	0.662496
		R	0.086135	0.874320	0.840365	0.856647	0.829742	0.847130
		R2	−1.339996	0.761948	0.702543	0.725385	0.682619	0.708490
	translation.z	RMSE	0.030548	0.023053	0.022737	0.023073	0.022583	0.040435
		MSE	0.000933	0.000531	0.000517	0.000532	0.000510	0.001635
		R	0.324380	0.803936	0.795007	0.811166	0.804379	0.709903
		R2	−7.316397	0.503342	0.516893	0.502510	0.523396	−0.527917
Slow walking	translation.x	RMSE	1.770301	0.499401	0.511289	0.498108	0.449248	0.515972
		MSE	3.133964	0.249401	0.261417	0.248111	0.201824	0.266228
		R	0.011000	0.921557	0.925347	0.917857	0.928084	0.906085
		R2	−0.268839	0.808397	0.799166	0.809388	0.844949	0.795470
	translation.y	RMSE	2.298411	1.000857	1.000378	0.990982	1.001029	1.178784
		MSE	5.282694	1.001714	1.000756	0.982046	1.002059	1.389532
		R	0.153591	0.861649	0.862975	0.863281	0.862232	0.807027
		R2	−4.294373	0.726944	0.727205	0.732305	0.726850	0.621229
	translation.z	RMSE	0.018717	0.005231	0.005327	0.005518	0.005566	0.013099
		MSE	0.000350	0.000027	0.000028	0.000030	0.000031	0.000172
		R	−0.130370	0.921210	0.917341	0.916645	0.913296	0.891483
		R2	−287.7802	0.845402	0.839658	0.827948	0.824970	0.030516

Subplots a, b, and c in Figures 8, 9 represent the comparison results of the position coordinates x, y, and z for different networks of the corresponding modes, respectively.

The evaluation indexes used in the experiments were root mean square error (RMSE), mean square error (MSE), correlation coefficient (R), and coefficient of determination (R2), and then the evaluation index EvaMe was obtained by a weighted averaging method:


(7) $$\text{EvaMe = }\frac{\sum_{\text{i}}^{\text{n}}\left(\alpha_{\text{i}}\times {\text{E}}_{\text{loss}}\right)}{\text{n}}$$

where *n* is the number of evaluation indicators, α_*i*_is the weight coefficient, *E*_*loss*_ is the evaluation indicator. Because the evaluation indicators selected in this paper are the error, the smaller the error, the higher the accuracy, and for the other two indicators used in this paper correlation coefficient and coefficient of determination is the closer to 1, the higher the accuracy if we want to use equation (7), will need to the correlation coefficient and coefficient of determination for error processing.

**Figure 10:**
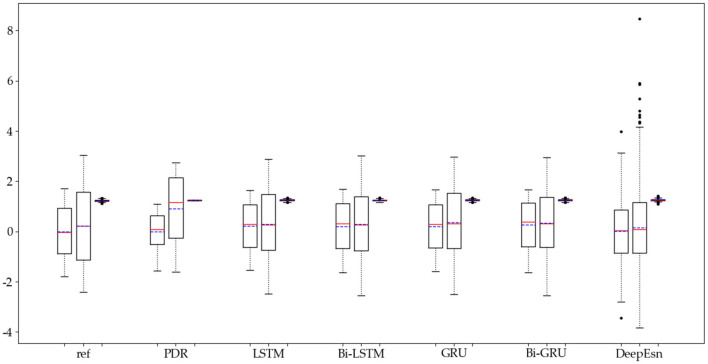
Distribution of location estimation results for different networks in the running mode.

**Figure 11:**
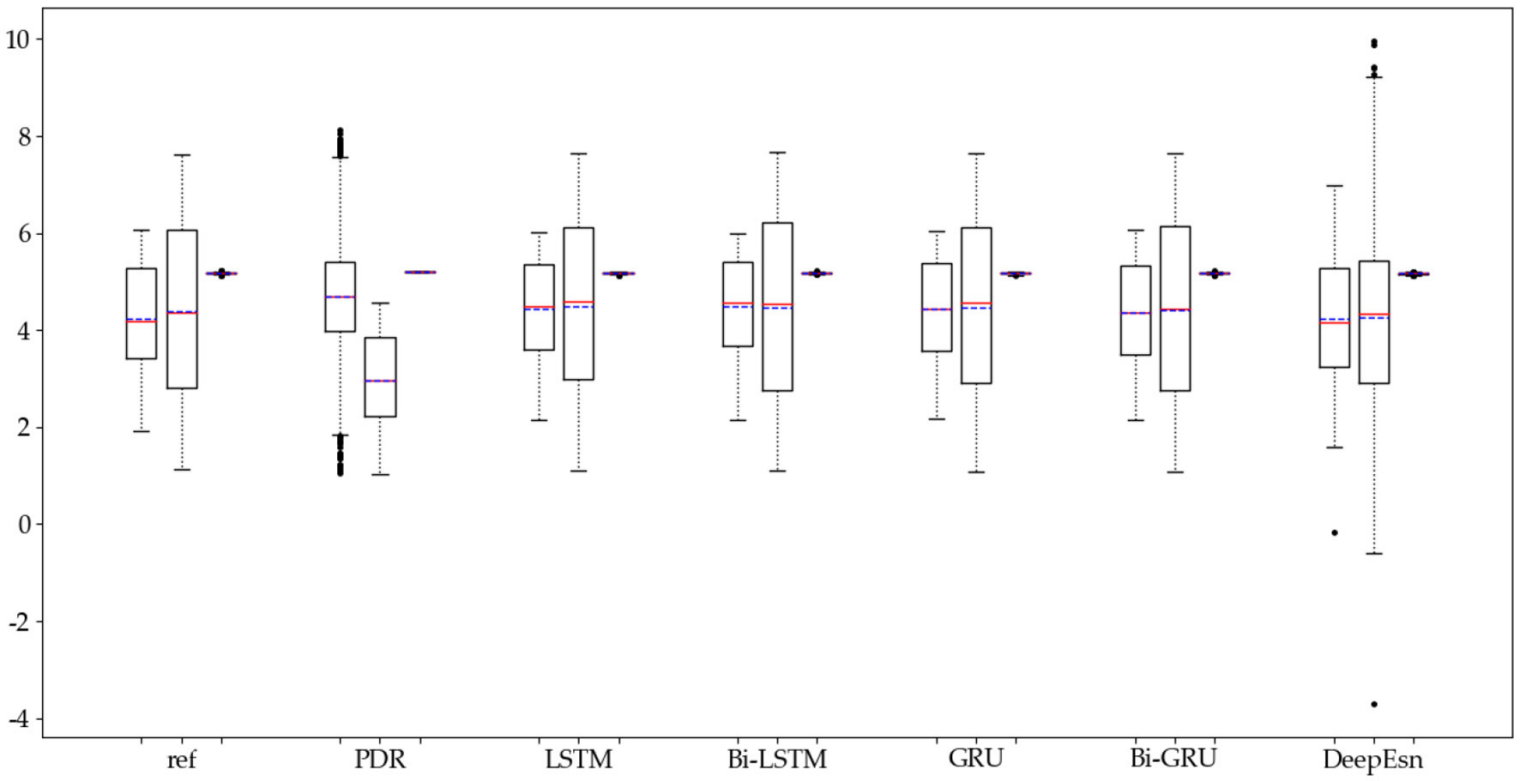
Distribution of location estimation results for different networks in the slow walking mode.

The structures selected for the DeepEsn networks in the running and slow walking modes are a 5-layer reserve layer with 600 neurons and a 6-layer reserve layer with 500 neurons, respectively. The evaluation metric EvaMe shows that the best prediction of position information is achieved by the LSTM network under running mode data. In contrast, the Bi-GRU network achieves the best prediction of location information under slow walking mode data. The distribution of data results predicted by each of its networks is shown in Figures 10, 11, and similar location information data are translated.x, translation.y, and translation.z from left to right.

**Figure 12:**
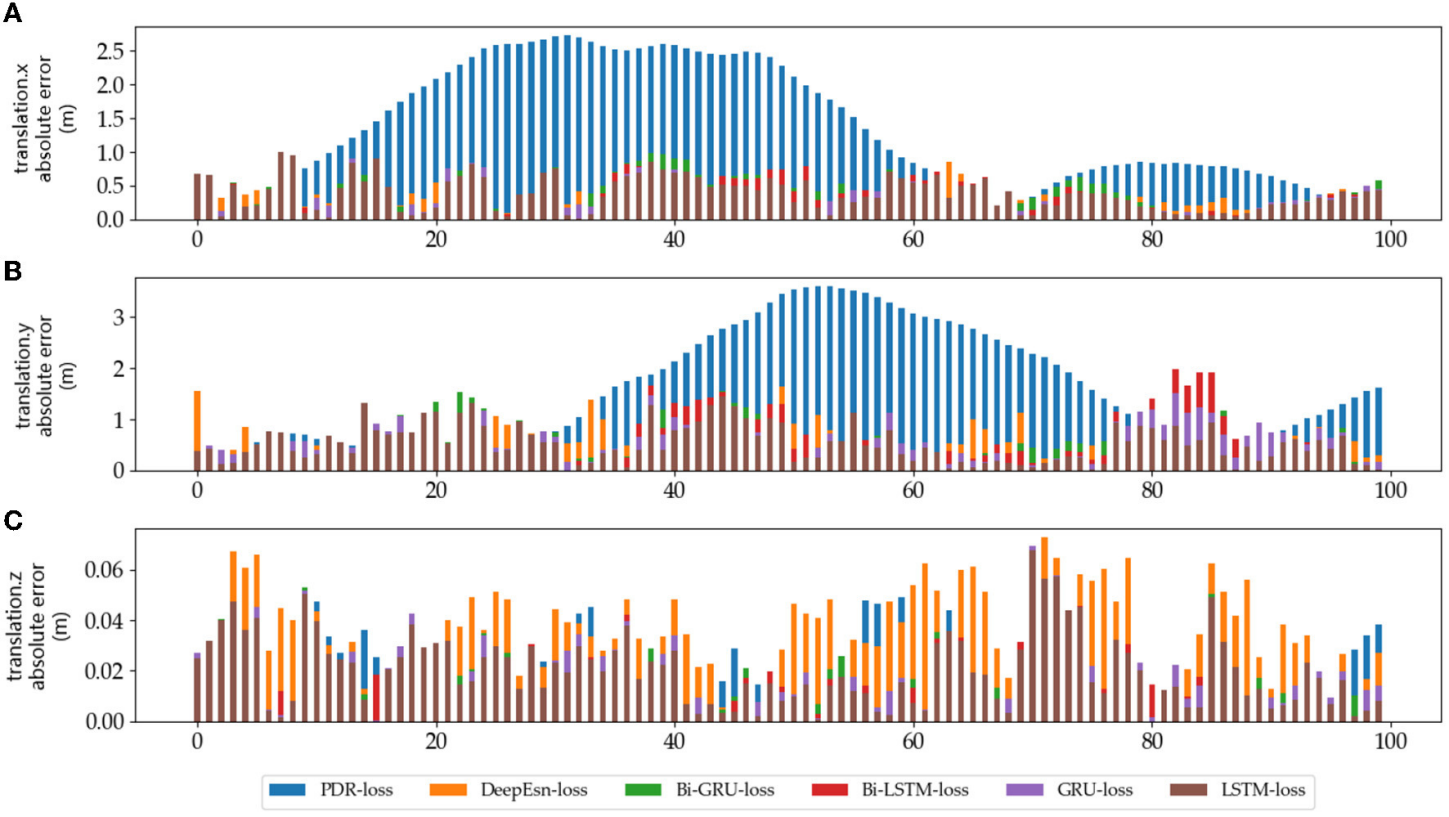
The absolute errors of different methods in running mode: **(A–C)** is the absolute errors of translation.x, translation.y, and translation.z in running mode, respectively.

**Figure 13:**
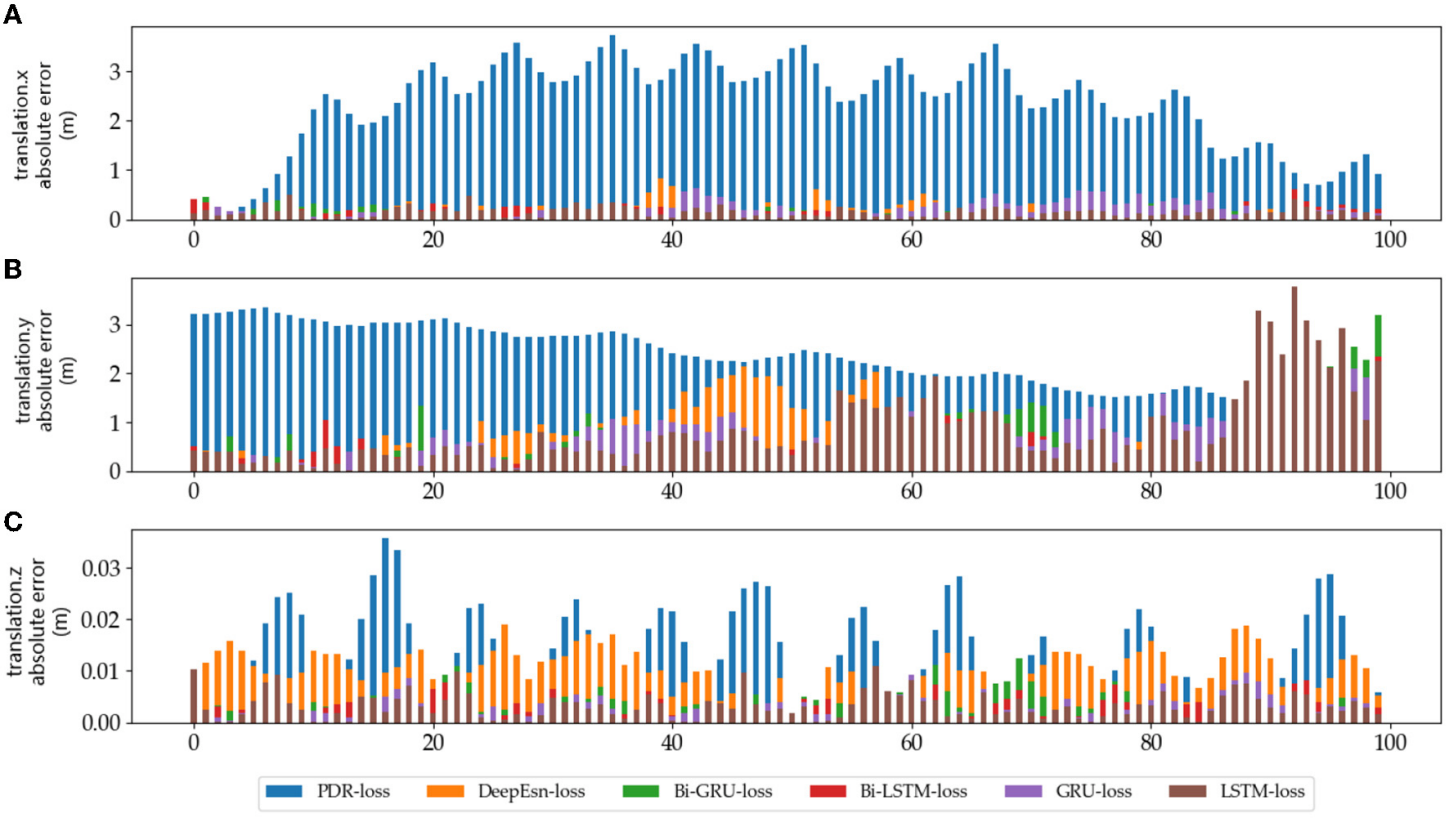
The absolute errors of different methods in slow walking mode: **(A–C)** is the absolute errors of translation.x, translation.y, and translation.z in slow walking mode, respectively.

The absolute error values of the predicted data results under different networks with reference data under running and slow walking mode data are shown in Figures 12, 13. The position absolute error plots are drawn by selecting 100 sets from the test set data, and the plots are translation.x, translation.y, and translation.z absolute error data.

Similarly, different deep networks estimate the location of handbag, handheld, pocket, and trolley mode data. The evaluation metrics EvaMe of their estimation results are shown in Table 6. The DeepEsn network structures in the table are the 5-layer 500 neuron reserve layer, 2-layer 500 neuron reserve layer, 7-layer 500 neuron reserve layer, and 5-layer 500 neuron reserve layer, respectively.

**Table 6:** Evaluation metric of EvaMe for each method in different modes.

**Mode**	**PDR**	**LSTM**	**Bi-LSTM**	**GRU**	**Bi-GRU**	**DeepEsn**
Handbag	3.85124	0.22663	**0.22525**	0.22822	0.23919	0.23812
Handheld	28.9174	1.13908	1.14954	1.10937	1.14782	**0.9371**
Pocket	49.0785	0.41175	0.4226	0.3961	0.39225	**0.29352**
Trolley	2.05988	0.33444	0.37399	0.36091	**0.33257**	0.38966

The bold values indicate the best evaluation metrics for each method in handbag, handheld, pocket, and trolley modes, respectively.

In Table 6, the estimation results indicate that the Bi-LSTM network should be selected for handbag mode. DeepEsn network of 2-layer and 500-neuron reserve should be selected for handheld mode. DeepEsn network of 7-layer and 500-neuron reserve should be selected for pocket mode. Bi-GRU network should be selected for trolley mode.

## 5. Discussion and conclusion

This paper proposes a method based on mode features and deep network matching to achieve location estimation. Firstly, feature extraction is performed on different mode data. The data's trend and detail features of the data are effectively extracted by discrete wavelet transform. Fourier transforms, and the data selection network is distinguished using mean, variance, kurtosis, and skewness mathematical indicators. Then, the classification is then performed by K-nearest neighbor and dynamic time regularization, and the classification accuracy reaches 90 from 30%, which shows the importance and necessity of data feature extraction methods. Finally, the evaluation indices of location information estimation under different modes prove the correctness and feasibility of the location estimation based on the matching method of mode features and deep networks. In this paper, the network is selected according to the decomposed mode features. The Bi-LSTM network is selected for the handbag mode, which has the trend cycle and small density amplitude. DeepEsn network is selected for the handheld mode with trend and detail cycles. The LSTM network is selected for the pocket mode with both the trend cycle and the detail cycle. And the LSTM network is selected for the running mode decomposition with a short trend cycle. The Bi-GRU network is selected for the running mode with a short trend period and dense detail. The DeepEsn network is selected after the slow walking mode decomposition with a long trend period and dense details. The Bi-GRU network is selected after the trolley mode decomposition with a long trend period. The sample entropy is used for the complexity of various mode types of data and the model's classification into categories. Many locational estimation experiments verify the feasibility of the method. Table 7. shows the network structure that should be selected for the different mode inputs.

**Table 7:** Network structures are selected for different mode inputs.

**Mode**	**Running**	**Slow walking**	**Handbag**	**Handheld**	**Pocket**	**Trolley**
Network	Bi-GRU	DeepEsn	Bi-LSTM	DeepEsn	LSTM	Bi-GRU
Layers	2	6	2	2	2	2
Number of neurons	32	500	32	500	32	32

The existing positioning and navigation techniques mostly use multiple positioning and navigation techniques to enhance the accuracy and application range of navigation and positioning through the fusion of advantages. At the same time, in this paper, we choose different adaptive depth networks for a single navigation technique with different input data modes to achieve positioning accuracy and expand the application range. In this paper, we choose different phase-adaptive depth networks to position and expand the application range. LSTM, GRU, Bi-LSTM, Bi-GRU, and DeepESN networks are more common deep networks with simple structures and parameters and easy-to-implement prediction functions. The end-to-end training approach relies on only one model and one objective function, which can circumvent the inconsistency in training multiple modules and the deviation of the objective function. The generalization can be obtained with the learning mode to solve the error accumulation problem in the traditional location solution. The model is more general for relying only on inertial data using a deep network model to estimate the position information. Only six modes are classified in this paper, and the mode types are limited. There is still a gap between the application scope and accuracy of single-location navigation and multi-positioning navigation techniques. Future work will solve these problems by replacing the adapted depth networks with more efficient and optimized neural networks and combining them with multi-location navigation techniques to improve accuracy.

## Data availability statement

The original contributions presented in the study are included in the article/supplementary material, further inquiries can be directed to the corresponding author.

## Author contributions

Y-TB: conceptualization. WJ and Y-TB: methodology and writing—review and editing. WJ, Y-TB, and X-BJ: writing—original draft preparation. Y-TB, X-BJ, J-LK, and T-LS: funding acquisition. All authors have read and agreed to the published version of the manuscript.
